# Horsford’s Acid Phosphate

**Published:** 1887-03

**Authors:** 


### HORSFORD'S ACID PHOSPHATE.

We are always gratified to learn that the remedies advertised in THE JOURNAL are efficacious in the treatment of disease. The following extract relating to Prof Horsford's "Acid Phosphate," amply justifies what is claimed for it as a remedial agent:

Cases of skin eruptions, etc., treated with Horsford's Acid Phosphate, by Mr. James Startin, late Honorary Surgeon and Lecturer, St. John's Hospital for Skin Diseases, London; Honorary Consulting Snrgeon to the Sheffield Public Hospital for Skin Diseases.

It appears to me that the "Acid Phosphate" originally prescribed by Prof. Horsford, of Cambridge, U. S. A., is not so well known in this country as its merits deserve. A glance at the formula will, however, readily convince one of its value in suitable cases. Each fluid drachm gives on analysis 5½ grains of free phosphoric acid, and nearly 4 grains of phosphate of lime, magnesia, iron and potash. The following are a few brief notes of some of the cases in which I have prescribed it with complete success.

Mr. G., æt. 69, consulted me November, 1885, for eczema on the arms, legs, palms of the hands and trunk. The patient complained of much debility and nervous exhaustion, and he was a man who had led a very busy business life, with much worry. In December, 1885, I prescribed Horsford's acid tonic with much good effect, and in February, 1886, I heard that hewas quite well.

Mrs. S., æt. 46, consulted me in December, 1885, for psoriasis, all over the body, more or less, especially on the legs and arms. In January, 1886, I prescribed a teaspoonful of the acid tonic three times a day with marked good effect. Patient had been much exhausted by continuous nursing on an invalid mother.

Mr. C., æt 64, consulted me in September, 1885, with one of the worst attacks of late syphilis I ever saw. After he had been relieved from the distressing symptoms, and ulcerations, I prescribed the acid tonic for epileptiform fits from which he suffered, with excellent results.

Mr. McJ., æt 63, consulted me, in November, 1885, for lichen ruber, which was accompanied with intolerable itching. He was a nervous, irritable man. I prescribed the acid tonic with the effect that in December he presented himself quite convalescent.—*Medical Press*, London, England.

---

RIO CHEMICAL CO. of ST. LOUIS, LONDON, PARIS — Some weeks ago we received from the above company, samples of medicine made by them, as advertised in this journal. After having observed the effects of these remedies, we are able to advisedly recommend them as among the very best of the kind obtainable. This opinion is backed by eminent practitioners in the leading cities of this country, who have given them practical tests.

